# Effect of increasing temperatures on duckweed growth, photosynthetic performance and frond ontogenesis

**DOI:** 10.1007/s00299-026-03943-1

**Published:** 2026-08-18

**Authors:** Muhammad Irfan, Muhammad Adil Chaudhry, Manuela Bog, Sándor Szabó, Ilona Mészáros, Marcel A. K. Jansen, Viktor Oláh

**Affiliations:** 1 School of Biological, Earth and Environmental Sciences, Sustainability Institute, University College Cork, Distillery Fields, North Mall, Cork, T23 TK30 Ireland; 2 Department of Botany, Institute of Biology and Ecology, Faculty of Science and Technology, University of Debrecen, Egyetem Square 1, 4032 Debrecen, Hungary; 3 Institute of Botany and Landscape Ecology, University of Greifswald, Soldmannstr. 15, 17489 Greifswald, Germany; 4 Department of Biology, Institute of Environmental and Natural Sciences, University of Nyíregyháza, 4401 Nyiregyhaza, Hungary

**Keywords:** Climate change, Global warming, Duckweed, Aquatic macrophytes, Chlorophyll fluorescence

## Abstract

**Key message:**

Duckweeds offer a suitable platform to study the effects of elevated temperatures on aquatic plants, and chlorophyll fluorescence imaging can reveal frond-level distribution of temperature stress.

**Abstract:**

Temperature rise associated with climate change poses a major challenge for aquatic plants. It has a strong influence on their physiological performance and, consequently, their competitive ability and fitness. Yet comparative studies of impacts on different species remain scarce. This study investigated the effects of four different temperature regimes between 20 and 35 °C on five duckweed species (*Spirodela polyrhiza*, *Landoltia punctata*, *Lemna gibba*, *Lemna minor* and *Lemna* *minuta*) by integrating growth- and photosynthesis-based parameters. We also recorded spatial patterns of photosystem II efficiency within fronds using chlorophyll *a* fluorescence imaging to understand how temperature stress is distributed throughout the frond development. The results highlighted substantial inter-specific variation in thermal responses and underscore the importance of integrating physiological and growth-based indicators to predict aquatic plants’ responses to climatic warming. Through simultaneous measurement of growth performance, photosynthetic efficiency and within-frond heterogeneity, this study provides mechanistic insights into how rising temperatures may influence duckweeds. Such knowledge is essential for predicting future shifts in freshwater primary productivity, species dominance, biological invasions and ecosystem stability under the current global warming scenario.

**Supplementary Information:**

The online version contains supplementary material available at https://doi.org/10.1007/s00299-026-03943-1.

## Introduction

Global warming has been causing a steady increase in ambient temperatures, and aquatic ecosystems are particularly facing rapid and pronounced warming (IPCC 2023). Especially, the water temperature in shallow water systems (10–20 m depth) including rivers, shallow lakes and ponds is directly linked to seasonal temperature variations as opposed to the geothermal temperature control present in deeper water layers (Kurylyk and Irvine 2019; Benz et al. 2024). An annual average temperature rise of up to 0.08 °C in riverine ecosystems has been reported across the globe (Liu et al. 2020). Similarly, global mean lake temperature was reported to have increased at a rate of 0.34 °C per decade between 1985 and 2009 (O’Reilly et al. 2015). Such a change in temperatures can predictably cause a widespread shift in photosynthetic limitations in plants (Sage and Kubien 2007). Therefore, freshwater aquatic plants, and particularly floating macrophytes, are highly relevant to predict the effects of global warming on shallow freshwater ecosystem stability and productivity (Koleszár et al. 2024).

The free-floating macrophyte family of duckweeds (Lemnaceae) is a natural inhabitant for shallow, slow-moving freshwater ecosystems like lakes, ponds, ditches and small streams (Ziegler et al. 2023). Despite their small size (1–15 mm), duckweeds are well known for being cosmopolitan and genetically diversified. To date, there are 36 recognised species of duckweeds within 5 genera (i.e. *Spirodela*, *Landoltia*, *Lemna*, *Wolffia* and *Wolffiella*), and multiple intra-specific clones within each species (Bog et al. 2019; Hoang et al. 2022). These small plants are responsible for substantial primary production, nutrient uptake and carbon cycling, particularly in shallow waterbodies (Landolt and Kandeler 1987; Ziegler et al. 2015). Moreover, they are used as model organisms for various research studies including anatomy, genomics, ecotoxicology and ecophysiology, i.e. for studying growth, physiological and environmental responses. Such a wide application is primarily due to their fast growth, reduced morphological complexity, easy maintenance and manipulation, presence of recognised stock collections and high inter- and intra-specific genetic diversity (Acosta et al. 2021). Currently, duckweed utilisation ranges from evolutionary studies and metabolomics to industrial applications (Appenroth et al. 2025). Due to ubiquitous distribution of Lemnaceae, and colonisation in different geographical and thermal niches, duckweeds display strong inter-specific adaptations to temperature changes, which might affect their competitiveness under the current global warming conditions (Mascheretti et al 2026). Hence, duckweed species are excellent candidates for studying implications of increasing temperatures in freshwater aquatic systems. In addition, the growing agricultural and industrial interest in duckweeds as prospective crops also necessitates better understanding of their acclimation to increased temperatures.

Generally, a temperature increase within plants’ optimum range stimulates growth but further increase beyond these levels results in reduced biomass production due to increased respiratory losses and impaired photosynthetic efficiency (Yamori et al. 2014). This overall growth impairment is a result of complex photosynthetic regulations, and measuring growth responses alone might not be enough to detect early-stage or sub-optimal stress effects, hence requires monitoring complementary physiological indicators. Photosynthesis and associated processes including water-splitting, electron transport, CO_2_ assimilation, photophosphorylation, chlorophyll biosynthesis, thylakoid membrane fluidity and photochemical reactions are very sensitive to heat stress (Hu et al. 2020; Rintoul et al. 2026). Chlorophyll *a* fluorescence (ChlF) is a non-destructive, in vivo technique for assessing functional efficiency of photosystem II (PSII), a complex site for photosynthetic processes located within thylakoid membranes of chloroplasts. ChlF has been widely used in studying plant stress physiology including environmental and toxicological responses, particularly in duckweeds (Maxwell and Johnson 2000; Oláh et al. 2021; Irfan et al. 2024; Szabó et al. 2025). Using ChlF, the proxies for PSII efficiency are generally calculated as F_v_/F_m_—known as maximum quantum yield in dark-adapted state, and Y(II)—known as the “Genty parameter” or actual efficiency of PSII in light-adapted steady-state photosynthesis (Genty et al. 1989; Roháček 2002).

Both these ChlF-based indicators, F_v_/F_m_ and Y(II), are widely used in ecophysiological stress studies as representatives of photoinhibition or damage to the photosynthetic apparatus (Guidi et al. 2019). F_v_/F_m_ commonly reflects photoinhibition or damage to the photosynthetic apparatus and its decline often precedes visible growth inhibition (Jiao and Hu 2025). On the other hand, Y(II) is closely linked to overall photosynthesis-related processes, e.g. water splitting, electron transport rates and carbon assimilation, and therefore it is highly sensitive to changing temperature or light conditions (Baker 2008). Together, these indicators can offer complementary insights into operational and potential photosynthetic changes under different temperatures. However, most duckweed studies have relied on ChlF measurements at the level of entire colonies or cultures, and, hence, only reported overall photosynthetic performance. With the recent advancements in ChlF imaging, it has become possible to record spatially resolved photosynthetic patterns in heterogeneous plant tissues such as within plant leaves and duckweed fronds (Baker 2008; Lahive et al. 2012). Recent evidence confirmed that disentangling smaller-scale patterns, such as responses of single fronds and within-frond regions, could facilitate understanding of duckweed environmental responses beyond colony levels (Oláh et al. 2024; Peršić et al. 2025; Xuan et al. 2025), thus generating new and deeper understanding of plant environmental responses.

In this study, we investigated the effects of four different temperatures on five duckweed species representing three genera by integrating growth-based and photosynthesis-based parameters. By comparing multiple species across variable temperature treatments, this study aimed to identify common and divergent physiological strategies underlying thermal responses in duckweeds and their potential implications for other aquatic plants. By studying spatial patterns of PSII efficiency within fronds, the study also aimed to understand how temperature stress is distributed throughout ontogenesis.

## Materials and methods

### Plant material

Axenic stocks of the five species (Table 1), i.e. *Spirodela polyrhiza* (L.) Schleiden (*S. polyrhiza*), *Landoltia punctata* (G.Mey.) Les & D.J. Crawford (*La. punctata*), *Lemna gibba* L. (*L. gibba*), *Lemna minor* L. (*L. minor*) and *Lemna minuta* Kunth (*L. minuta*) were maintained in 100 ml Erlenmeyer flasks using 50 ml of the modified Steinberg medium—see OECD (2006) for composition—under continuous white light (PPFD 60 ± 10 μmol m^−2^ s^−1^) and controlled temperature of 24 ± 2 °C. These species were chosen for the study because of both their economic and ecological relevance: *S. polyrhiza*, *La. punctata*, *L. gibba* and *L. minor* are amongst the most studied duckweed species for biomass production and phytoremediation. Besides, *S. polyrhiza*, *L. gibba* and *L. minor* are cosmopolitan, inhabiting a variety of biogeographical regions worldwide. *Lemna minuta*, on the other hand, had only been recently introduced to Europe and Asia, and *La. punctata* is also considered to be a neophyte to both European and North American flora (Tippery and Les 2020). Their thermotolerance, hence, may affect their competitiveness in newly colonised habitats.

**Table 1:** Photographs and details of five duckweed species used in experimental work

Species	Clone ID	Origin	Photograph
*S. polyrhiza*	UD0403	Szarvas, Hungary	
*La. punctata*	Landolt clone #7760	Caroline Sinkhole, Australia	
*L. gibba*	UD0106	Hajdúbagos, Hungary	
*L. minor*	UD0216	Miskolctapolca, Hungary	
*L. minuta*	UD0303	Copenhagen, Denmark	

The clones were subsequently subcultured and grown in 300 ml Erlenmeyer flasks using 100 ml of the modified Steinberg medium for 7 days before the experiments.

### Experimental design

The growth experiments were conducted under controlled light and temperature conditions using an indoor plant growth chamber (FH-721, HiPoint, Taiwan). A single healthy colony (3–4 fronds) of the respective duckweed species was placed in each well of the tissue culture plates (6-well plates, 9.60 cm^2^ area per well) and supplied with 12 ml of modified Steinberg medium. Duckweed growth was measured over 5 days using four different temperature treatments (i.e. 20, 25, 30 and 35 °C) under continuous light (PPFD ~56 μmol m^−2^ s^−1^; daily light integral of 4.8 mol m^−2^) consisting of ~1:1 mix of blue (452 nm) and red light (657 nm). Though such an irradiation regime did not mimic natural scenarios, constant low-intensity illumination was chosen to ensure rapid growth under standardised conditions whilst decreasing the risk of photoinhibition under either temperature regime. Previous literature indicated that photosynthetic light saturation of *L. minor* and *S. polyrhiza* took place between 300 and 1200 μmol m^−2^ s^−1^ when studied as a function of ambient temperature from 15 to 35 °C (Wedge and Burris 1982). Similarly, growth of *Lemna* species saturated at ~15 mol m^−2^ with no significant growth gain occurring above 7.5 mol m^−2^ (Pasos-Panqueva et al. 2024).

The experiments were repeated twice with three internal replicates at each temperature setting under identical conditions.

### Chlorophyll fluorescence imaging and growth measurement protocol

ChlF imaging was performed using a Maxi Imaging-PAM fluorometer (Heinz Walz GmbH, Effeltrich, Germany) on the initial (0th) and final (5th) days of the growth experiments. Following the protocol by Irfan et al. (2024), plants were directly transferred to the measuring chamber of the instrument and measured for light-adapted parameters F_s_ and F_m_′ after 60 s of actinic illumination (~ 56 μmol m^−2^ s^−1^). The dark-adapted parameters, i.e. F_o_ and F_m_, were then separately measured after placing the plants in dark for 20 min. All parameters were averaged on a well-basis and used to calculate the maximum quantum yield of PSII in the dark-adapted state, i.e. F_v_/F_m_, and the actual quantum yield of PSII under ambient conditions, i.e. Y(II), according to the following formulas (Roháček 2002):

$${F}_{v}/{F}_{m}=({F}_{m}-{F}_{o})/{F}_{m}$$

$$Y(II)=\left({F}_{m}{\prime}-{F}_{s}\right)/{F}_{m}{\prime}$$

Growth parameters including the total number of fronds and colonies and their corresponding total surface area on the initial and final days were measured by means of F_m_ images in ImageJ software (v1.54 g) environment, plugins: “Cell counter”, “Threshold colour” and “Analyze particles” (Abramoff et al. 2004; Schneider et al. 2012), refer to Irfan et al. (2024) and Oláh et al. (2023) for detailed protocols. These parameters were then used to calculate duckweed growth indicators including relative growth rate (RGR) and colony size (OECD 2006; Oláh et al. 2024) as follows:

$$RGR = (\mathrm{ln}\left({SA}_{5}\right)- \mathrm{ln}\left({SA}_{0}\right))/t$$

$$Colony size ={N}_{f}/{N}_{c}$$

where SA_5_ and SA_0_ represent the total frond surface area on 5th and 0th days, respectively, t corresponds to the growth duration (i.e. 5 days), N_f_ is the total number of fronds and N_c_ is the total number of colonies on the final day of experiments. Additionally, average frond area was also calculated using total surface area and number of fronds on the 5th day of experiments:

$$Avg. frond area ={SA}_{5}/{N}_{f}$$

### Measurement of within-fronds ChlF patterns

In addition to measuring well-averaged values of F_v_/F_m_ and Y(II), the greyscale ChlF images (256-bit jpg) taken on the 5th day of experiments were exported using ImaginWinGigE software and analysed for within-fronds patterns following the protocol by Oláh et al. (2024). Briefly, for both F_v_/F_m_ and Y(II) images, relative pixel densities (0–255) with their corresponding pixel number were recorded along a linear transect (base to apex) on individual duckweed fronds using the “plot profile” function in ImageJ software (Oláh et al. 2024). The produced dataset was further processed to convert the pixel densities back to the respective quantum yields, i.e. F_v_/F_m_ or Y(II), considering their theoretical ranges to be between 0 and 1. The dataset was also used to calculate individual frond length using the resolution-derived pixel size (∼0.22 mm; 4.6-pixel mm^−1^) in the applied experimental setup. The following formulas were used for these conversions (Oláh et al. 2024).

$$\Phi =\left(1/256\right)\times {I}_{px}$$

where Φ represents quantum yield in terms of F_v_/F_m_ or Y(II) and I_px_ corresponds to relative pixel intensity.

$${L}_{f}= {N}_{px}\times 0.22$$

where L_f_ is the total frond length in mm and N_px_ is the total number of pixels along the transect.

### Data analysis and statistics

The data collected from two separate experiments were combined to produce a total n=6 for each treatment. Individual and interactive effects of species and temperature regimes were evaluated by means of two-way ANOVA performed in RStudio (2024.12.1+563, RStudio Team 2020). Descriptive statistics and one-way ANOVA with Tuckey’s HSD post hoc tests were also performed in RStudio at a significance level of 95% (*p*<0.05). Different software environments were used for graphing work, including OriginPro 2016 (v b9.3.226) for bar plots, MS Excel (v2601) for regression plots, RStudio for kernel density plots, ‘akima’ (Akima and Gebhardt 2022) and ‘fields’ (Nychka et al. 2021) packages in RStudio for within-fronds pattern interpolations. In the latter plots, median values of pixels in overlapping positions were used for interpolations. Ordinary least squares linear regression models describing frond-averaged photochemical efficiency as functions of frond length were fitted by means of Past v4.0 (Hammer et al. 2001). This latter software was also used to compare the equality of slopes of the fitted models.

## Results

### Growth performance

**Fig. 1:**
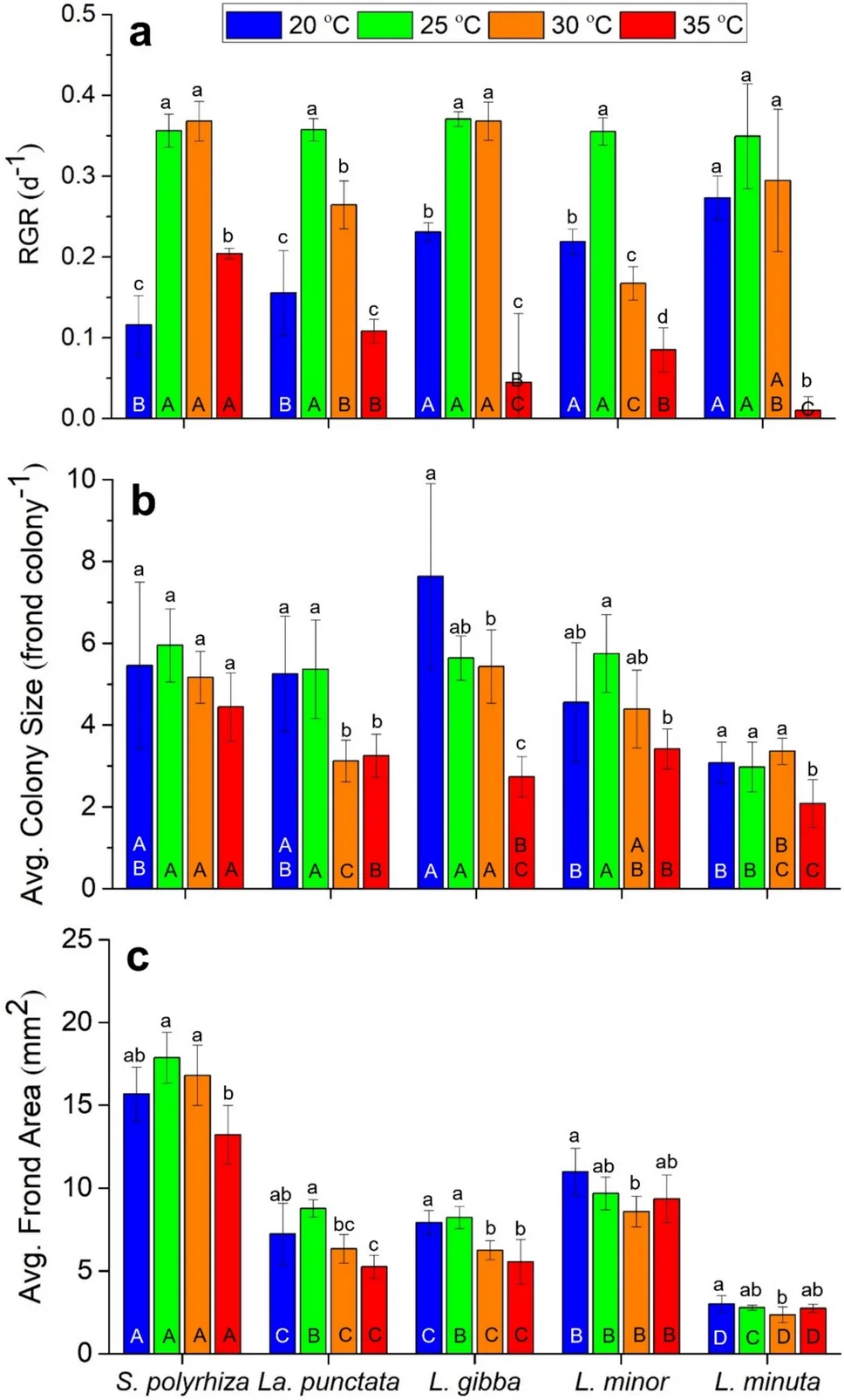
Measured values (means of n = 6 ± SD) of growth parameters: **a** relative growth rate (RGR), **b** average colony size and **c** average frond area of five duckweed species, *S. polyrhiza*, *La. punctata*, *L. gibba*, *L. minor* and *L. minuta*, under different ambient temperatures. Different lower-case letters above the bars (a, b, c) denote statistically significant differences (*p* < 0.05) of the same species across different temperatures, whereas different capital letters at the base of the bars (A, B, C) denote statistically significant differences (*p* < 0.05) amongst different species at the same temperature, according to one-way ANOVA and post hoc Tuckey’s HSD tests. The lower-case letters should be compared only within each group of bars, and the capital letters should be compared between the bars of the same colour

All tested duckweed species showed the highest RGRs at 25 °C with an overall average value of 0.35 ± 0.03 d^−1^*.* Two-way ANOVA showed significant effects of both factors; i.e. species, temperature as well as their interaction (*p* <0.001 for all, detailed results are provided in Supplementary Table S1) on the RGR, colony size and frond area. *Lemna gibba* and *S. polyrhiza* had similar RGR values at 30 °C, significantly outperforming *La. punctata* and *L. minor*. Due to variable response, RGR of *L. minuta* remained comparable to that of *L. gibba*, *S. polyrhiza* and *La. punctata* at 30 °C. RGR for *L. gibba*, *L. minor* and *L. minuta* were significantly higher than those of *S. polyrhiza* and *La. punctata* at 20 °C. On the other hand, *S. polyrhiza* showed significantly greater RGR values at 35 °C and both *L. minor* and *La. punctata* had statistically similar and comparatively lower RGRs than *S. polyrhiza*. No statistically significant differences were found between growth of *L. gibba*, *L. minuta* and *S. polyrhiza* between 25 and 30 °C treatment groups, whilst *L. minor* and *La. punctata* showed a significant drop in RGRs at 30 °C. The lowest RGR values within each species were recorded at 35 °C except for *S. polyrhiza* at 20 °C (Fig. 1a).

Colony size amongst the tested duckweed species also showed variations and the largest colony size was recorded at 25 °C for all the tested species except for *L. gibba* which produced larger colonies at 20 °C, though the difference was non-significant compared to 25 °C. The smallest colony size, on the other hand, was recorded at 35 °C except for *La. punctata* where comparable-sized colonies were produced under both 30 and 35 °C. Except for *L. gibba*, an overall non-significant increase in colony size at 25 °C was recorded as compared to 20 °C, which then continued to drop with the increase in temperatures (Fig. 1b).

The results also showed changes in average frond area between different species and temperature treatments. Most of the tested duckweed species produced larger fronds at 25 °C except *L. minor* and *L. minuta* producing larger fronds at 20 °C. Smaller fronds, on the other hand, were produced at 35 °C, except for *L. minor* and *L. minuta* producing comparatively smaller fronds at 30 °C. Lowest variation in frond area across different temperatures was recorded for *L. minor* (CV = 14.89%) and *S. polyrhiza* (CV = 14.92%) whilst the greatest variability was present in *L. minuta* (CV = 51.9%, Fig. 1c).

**Fig. 2:**
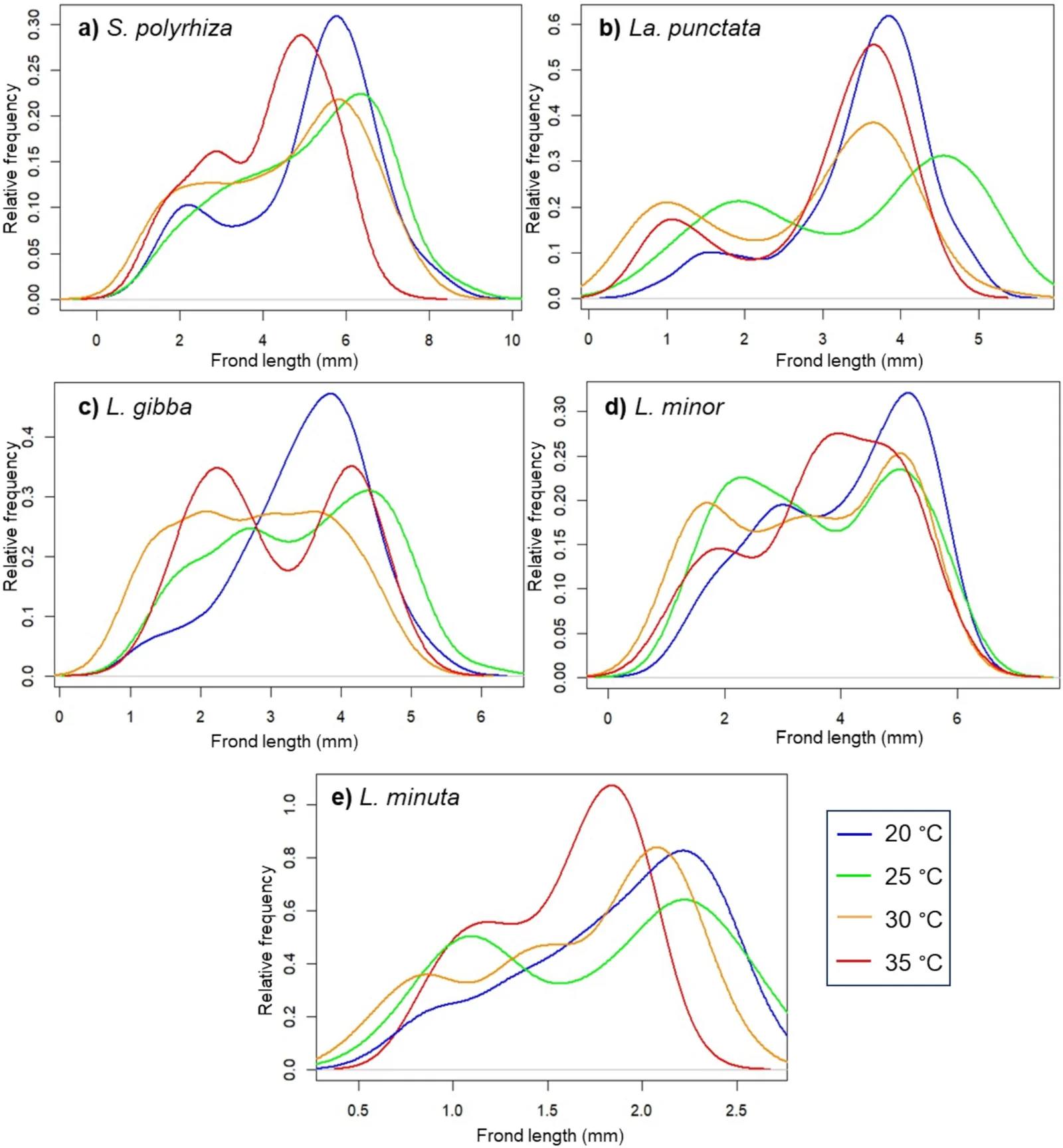
Kernel density plots showing relative frequency of fronds according to their length amongst the tested duckweed species: **a** *S. polyrhiza*, **b** *La. punctata*, **c** *L. gibba*, **d** *L. minor* and **e** *L. minuta* under different ambient temperatures. See Supplementary **Table S2** for frond length distribution summary statistics

Changes in frond length (base to apex) distribution patterns were also recorded under different temperature treatments. The tested species produced the longest mature fronds under 25 °C, followed by 20, 30 and 35 °C (Fig. 2a-e). The relative frequency (proportion) of longer (mature) fronds was comparatively higher than that of the smaller (younger) fronds in all the species across different temperature treatments, resulting in negatively skewed frond size distribution except *L. gibba* between 30 and 35 °C (Fig. 2c, Supplementary Table S2).

### Photosynthetic efficiency, frond ontogenesis and within-fronds patterns

**Fig. 3:**
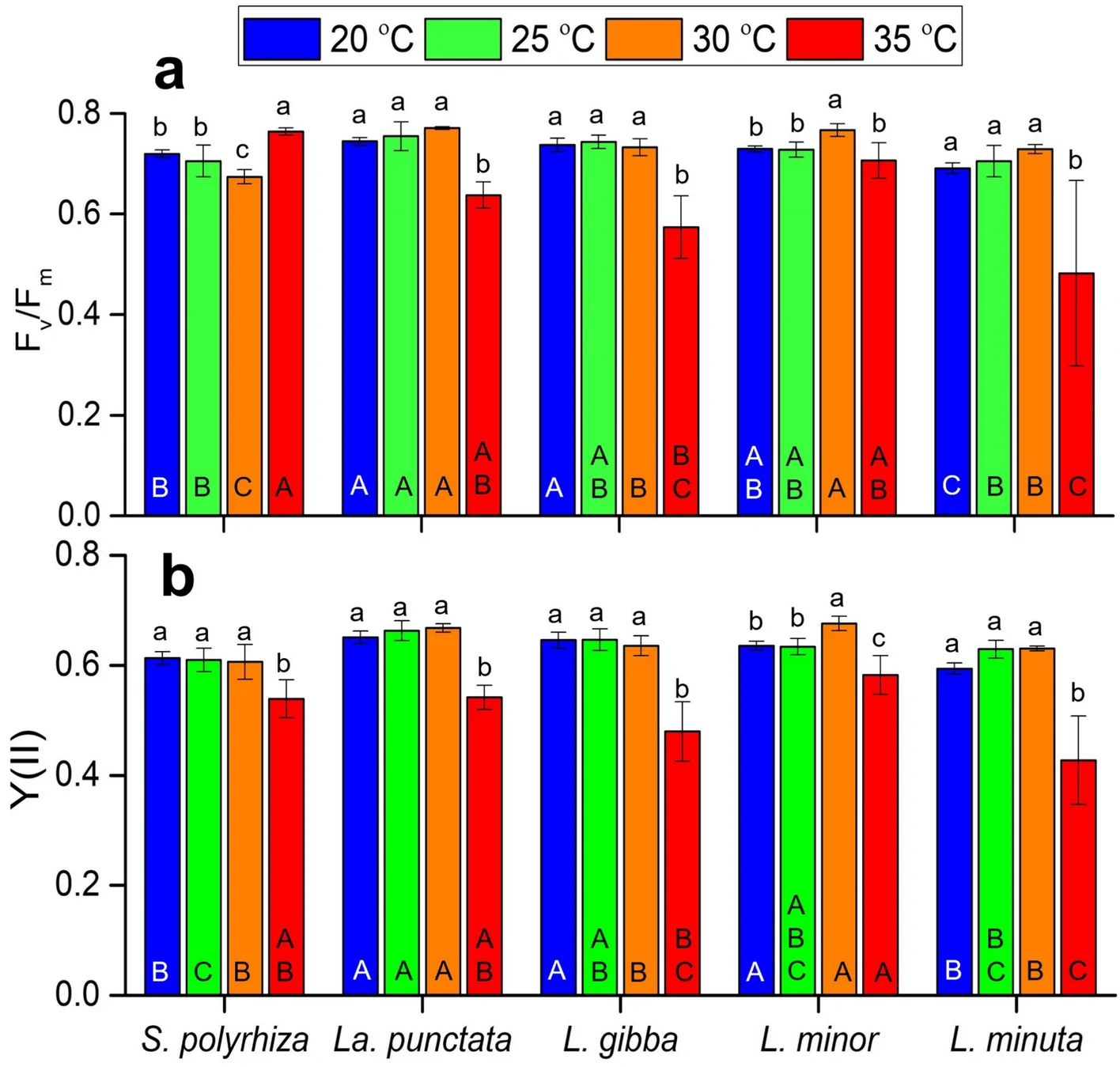
Measured values (means of n = 6 **±** SD) of ChlF parameters: **a** F_v_/F_m_ and **b** Y(II) for the tested duckweed species, *S. polyrhiza*, *La. punctata*, *L. gibba*, *L. minor* and *L. minuta*, under different ambient temperatures. Different lower-case letters above the bars (a, b, c) denote statistically significant differences (*p* < 0.05) of the same species across different temperatures, whereas different capital letters at the base of the bars (A, B, C) denote statistically significant differences (*p* < 0.05) amongst different species at the same temperature, according to one-way ANOVA and post hoc Tuckey’s HSD tests. The lower-case letters should be compared only within each group of bars, and the capital letters should be compared between the bars of the same colour

When the results for F_v_/F_m_ and Y(II) were compared using two-way ANOVA, significant effects of species, temperature and their interaction (p <0.001 for all, detailed results provided in supplementary Table S1) were observed. The tested duckweed species, however, showed no significant differences in the mean F_v_/F_m_ and Y(II) in response to different temperatures from 20 to 30 °C, except for a few instances (Fig. 3a, b). Significantly lower values for Y(II) were recorded across the species at 35 °C as compared to lower temperature treatments (Fig. 3b). F_v_/F_m_, on the other hand, showed different inter-specific patterns, e.g. F_v_/F_m_ of *S. polyrhiza* was significantly higher at 35 °C than at 20, 25 and 30 °C. *L. gibba* and *L. minuta* showed significantly lower F_v_/F_m_ and Y(II) values at 35 °C compared to lower temperatures. Overall, *La. punctata* showed higher F_v_/F_m_ and Y(II) values between 20 and 30 °C compared to other species. Relatively higher values for both F_v_/F_m_ and Y(II) were also recorded for *S. polyrhiza*, *La. punctata* and *L. minor* at 35 °C whilst *L. minuta* had the lowest photochemical efficiency.

**Fig. 4:**
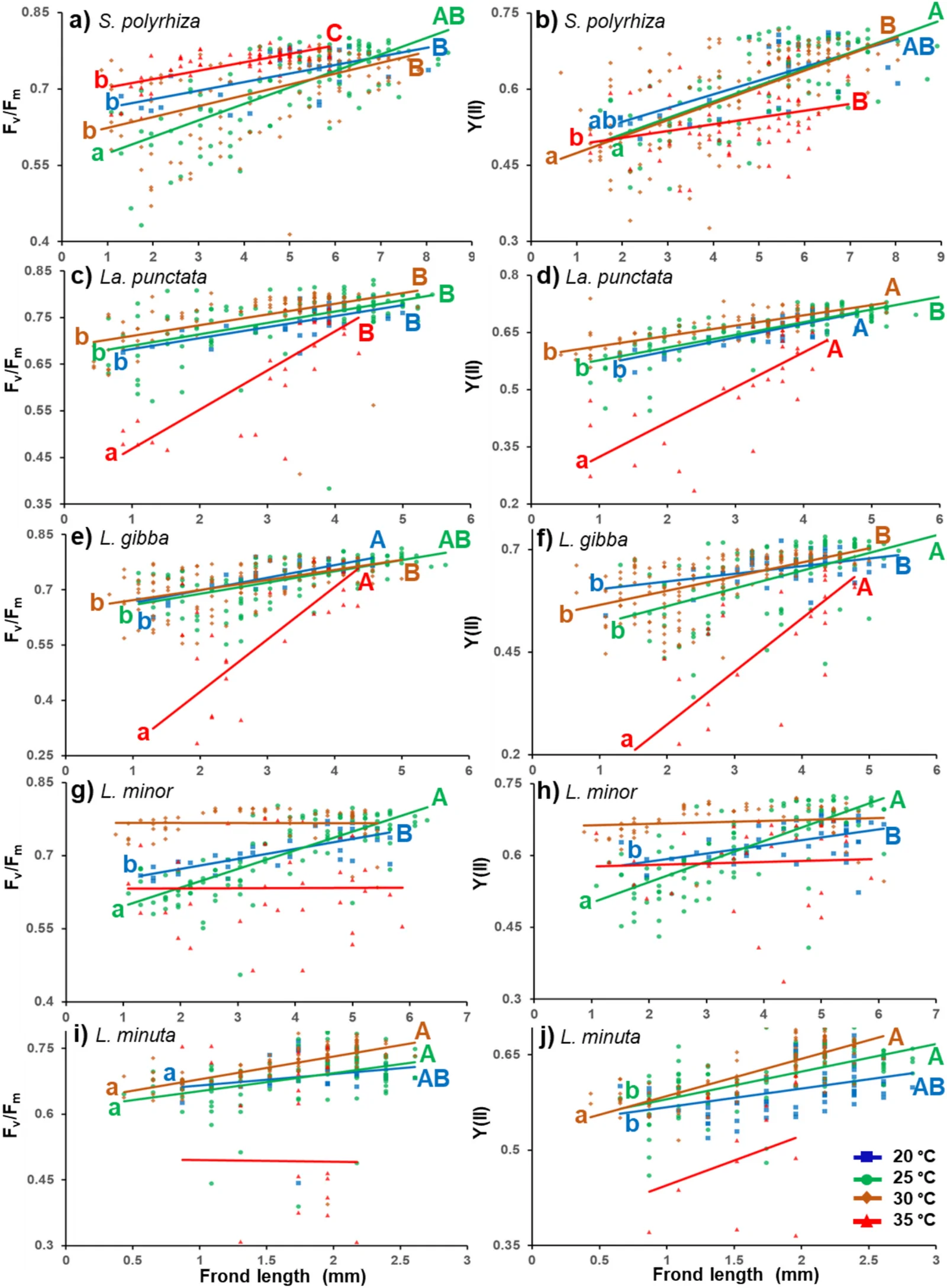
Variability in dark-adapted (F_v_/F_m_, subfigures **a**, **c**,** e**, **g** and **i**) and light-adapted photosynthetic efficiency (Y(II), subfigures **b**, **d**, **f**, **h** and **j**) of tested duckweed species **a–b** *S. polyrhiza*, **c–d** *La. punctata*, **e–f** *L. gibba*, **g–h** *L. minor* and **i–j** *L. minuta* as a function of frond ontogenetic stage under different ambient temperatures. Different lower-case letters (a, b, c) denote statistically significant differences (*p* < 0.05) of the regression model slopes for the same species across different temperatures (i.e. within the same subfigure). Conversely, different capital letters (A, B, C) denote statistically significant differences (*p* < 0.05) amongst different species at the same temperature for F_v_/F_m_ (i.e. across subfigures **a**, **c**,** e**, **g** and **i**) or Y(II) (i.e. across subfigures **b**, **d**, **f**, **h** and **j**). Note: slopes of non-significantly (*p* > 0.05) fitting regression models were excluded from comparisons, hence lack both lower-case and capitals letters. Parameters of the fitted linear regression models, as well as pairwise comparisons of their slopes are reported in Supplementary Table S3

The photochemical efficiency of fronds at different ontogenetic stages (based on length) varied amongst different species and temperature treatments (Fig. 4a-j, Table S3). At 25 °C, the tested duckweed species showed a steady increase in ChlF efficiencies that was aligned with frond length with no significant difference (*p* > 0.05) in the steepness of the slope amongst species except for *La. punctata* (Fig. 4c, d). The younger fronds of *S. polyrhiza* showed similar F_v_/F_m_ and Y(II) values at 35 °C compared to the mature fronds. The overall F_v_/F_m_ values for *S. polyrhiza* were higher at 35 °C when compared to lower temperatures whilst Y(II) stayed comparatively lower at 35 °C (Fig. 4a, b). Shorter fronds at early ontogenetic stages in *La. punctata* and *L. gibba* showed lower F_v_/F_m_ and Y(II) values at 35 °C compared to longer fronds and to the fronds of the same size at lower temperatures. Longer fronds of *La. punctata* and *L. gibba*, on the other hand, reached similar ChlF efficiency at 35 °C to those at lower temperatures (Fig. 4c-f). Both young and mature fronds in *L. minor* and *L. minuta* showed similar ChlF efficiency to each other at higher temperatures, i.e. under 30–35 °C (Fig. 4g-j). Younger fronds of *L. minor* could maintain higher values for F_v_/F_m_ and Y(II) at 30 °C than the similar-sized fronds at lower temperatures, whilst the longer (mature) fronds displayed lower efficiencies at 35 °C compared to fronds with similar length but under lower temperature treatments (Fig. 4g, h). The maximum measured frond lengths and ChlF efficiencies for *L. minuta* at 35 °C were considerably lower as compared to those at the lower temperatures (Fig. 4i, j).

**Fig. 5:**
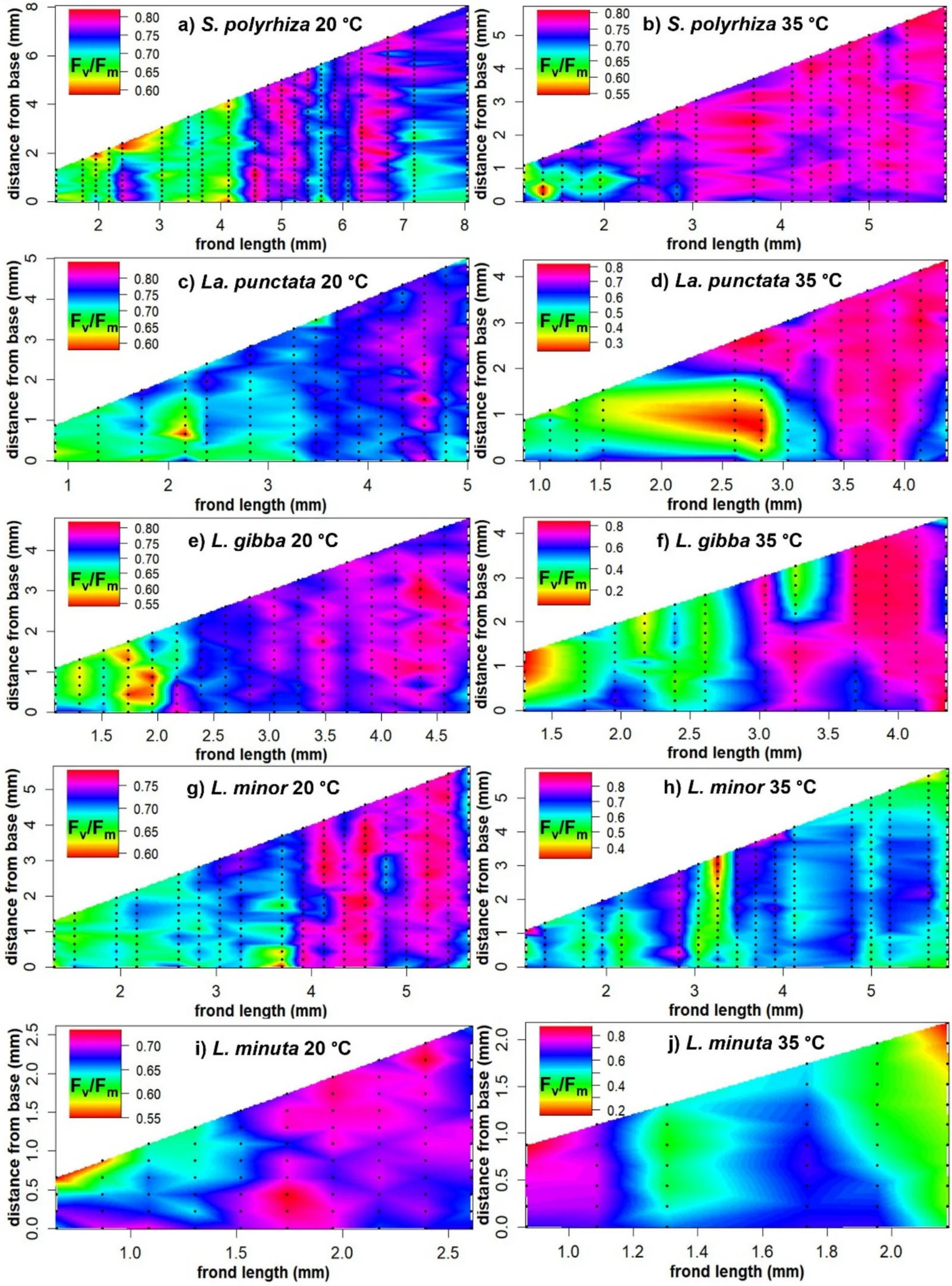
Within-frond patterns in dark-adapted photosynthetic efficiency (F_v_/F_m_) of tested duckweed species **a–b)** *S. polyrhiza*, **c–d)** *La. punctata*, **e–f)** *L. gibba*, **g–h)** *L. minor* and **i–j)** *L. minuta* for different frond ontogenetic stages under 20 and 35 °C ambient temperatures. The interpolation plots represent pixel-by-pixel F_v_/F_m_ values of different frond sizes along the horizontal axis and within-frond measured position from the frond base along the vertical axis. Black dots denote positions of the actually measured pixels along the transects. Note: The colour gradients were set separately for each plot

**Fig. 6:**
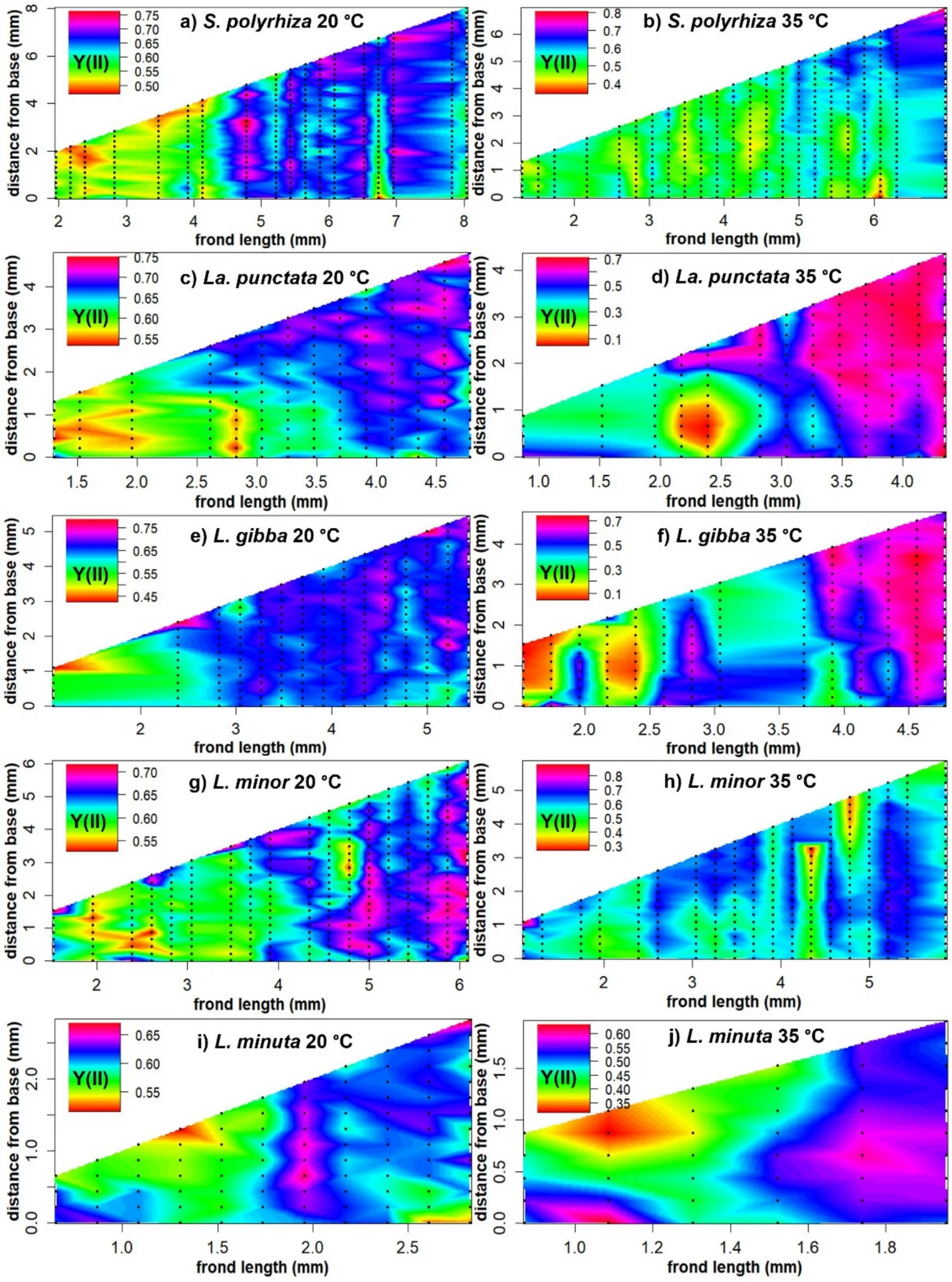
Within-frond patterns in light-adapted actual photosynthetic efficiency Y(II) of tested duckweed species **a–b)** *S. polyrhiza*, **c–d)** *La. punctata*, **e–f)** *L. gibba*, **g–h)** *L. minor* and **i–j)** *L. minuta* for different frond ontogenetic stages under 20 and 35 °C ambient temperatures. The interpolation plots represent pixel-by-pixel Y(II) values of different frond sizes along the horizontal axis and within-frond measured position from the frond base along the vertical axis. Black dots denote positions of the actually measured pixels along the transects. Note: The colour gradients were set separately for each plot

When the measured values for ChlF efficiencies F_v_/F_m_ and Y(II) were plotted against their position within the fronds, different distribution patterns emerged for the tested duckweed species under different ambient temperatures (Figs. 5a-j, 6a-j, supplementary Figs. S1, S2). It was noticed that the fronds could gain or lose photosynthetic maturity only after reaching a minimum threshold length (base to apex) depending on the species and temperature. A gradual increase in this frond threshold length was recorded from lower (20 °C) to higher ambient temperature (35 °C) for *L. gibba*, *L. minor* and *La. punctata*, whilst *L. minuta* and *S. polyrhiza* showed somewhat different patterns. In the case of *S. polyrhiza*, shorter frond threshold length was needed to reach F_v_/F_m_ maturity at 35 °C as compared to 20 °C (Fig. 5a, b). However, this threshold length increased in case of Y(II), and the fronds could not gain maximum Y(II) until they reached > 5 mm in length (Fig. 6a, b). In the case of *L. minuta*, that had the smallest fronds amongst the tested species, only fronds reaching ~1.5 mm length gained mature F_v_/F_m_ at 20 °C (Fig. 5i). Juvenile fronds (< 1.1 mm) at 35 °C showed higher F_v_/F_m_ values from base to apex than the mature fronds (Fig. 5j). The younger fronds (< 1.1 mm) of *L. minuta* also showed higher Y(II) along their basal parts than the distal parts, though mature fronds still retained higher Y(II) (Fig. 6i, j). Based on Y(II), the maturation of fronds initially started at the distal part of the fronds and moved gradually towards the base.

## Discussion

### Effects of temperature on duckweed growth

Duckweeds are well known for their plasticity and resistance to a wide range of environmental and anthropogenic stressors (Ziegler et al. 2015, 2023). They can efficiently trade-off between their growth, biochemical composition and photosynthetic performance to gain better environmental adaptability (Demmig-Adams et al. 2022). Due to these characteristics and presence of high genetic inter- and intra-specific variations, different duckweed species and clones often respond differently to external stress factors (Oláh et al. 2023; Xuan et al. 2025). These key characteristics make duckweeds excellent model organisms for environmental and toxicological response studies (Thingujam et al. 2024). In the present study, five duckweed species showed highest growth rates at 25 °C ambient temperatures with no significant differences in optimum temperature observed between the tested species. Previously published data also support that most duckweed species achieve optimum growth rates at similar temperatures under controlled conditions (Pasos-Panqueva et al. 2024). The growth rates for each tested duckweed species at 25 °C were comparable to previously reported values (Ziegler et al. 2015).

Diverging, temperature-dependent responses in growth rates were recorded between tested species. For instance, *L. minor* grew faster at lower temperatures and *S. polyrhiza* at higher temperatures**.** Growth response of *L. minor* was consistent with its geographical distribution as it is a low-to-moderate temperature species spread across Europe and Ural Mountains (Volkova et al. 2024). Better growth of *S. polyrhiza* at higher temperatures and slower growth at lower temperatures were also reported in previous studies (Appenroth 2002; Song et al. 2006). In addition, our results showed that *L. minuta* displayed higher RGRs between 20 and 30 °C than *L. minor*. This aspect highlights the importance of forecasting competitive abilities of recently spreading duckweed species under warming scenarios. *Lemna minuta* is considered invasive in Europe, and a strong competitor of native *L. minor* (Paolacci et al. 2018). Draga et al. (2024) analysed the distribution of alien aquatic plants in Poland, and found a strong association of *L. minuta* to higher yearly minimal temperatures. In addition, at elevated ambient temperature (30/25 °C day/night), *L. minuta* clones displayed faster growth and lower temperature sensitivity than *L. minor* did when compared to mild (20/16 °C day/night) conditions (Mascheretti et al. 2026). The same study also reported comparable or even higher RGR of other alien duckweed species in Europe such as *La. punctata* and *Lemna aequinoctialis* when compared to the native ones (Mascheretti et al. 2026). Hence, milder winters and prolonged occurrence of 20–30 °C temperatures in European lentic habitats might promote further spread of these species at higher latitudes.

Colony size and average frond area are often studied as stress proxies in duckweeds where both smaller colonies (lower number of fronds per colony) and fronds are produced under unfavourable or toxic conditions (Ziegler 2025). Our results for these stress proxies showed an association with slower overall growth rate. Thus, at temperatures above 20–30 °C, lower growth rates and smaller colonies and fronds were noted. Going hand in hand with frond surface area measurements, fronds were also recorded to be longer between 20 and 25 °C as compared to at 35 °C. These results are consistent with the previously reported optimum temperature ranges for duckweed species (Ziegler et al. 2023).

### Effects on photosynthetic performance and frond ontogenesis

The non-invasive ChlF-based indicators stayed in stable ranges of ~0.7 (F_v_/F_m_) and ~0.6 [Y(II)] between 20 and 30 °C. These values were comparable to previously reported control values of F_v_/F_m_, i.e. ~0.7 (Audenaert et al. 2026) and Y(II), i.e. 0.63 (Irfan et al. 2024). Interspecific differences in growth and ChlF performance can be hypothesised to be due to species-specific trade-offs (Ishizawa et al. 2021). Significantly lower values of F_v_/F_m_ and Y(II) occurred at 35 °C, indicating a drop in the photosynthetic performance of the tested duckweed species. Similar decreases in photosynthetic efficiency have been reported in the terrestrial plant species *Setaria viridis* at ambient temperatures between 31 and 40 °C (Anderson et al. 2021).

Whilst averaged F_v_/F_m_ and Y(II) at well level did not differ significantly amongst the tested duckweed species across different temperature treatments, within-frond ChlF values revealed interesting patterns for frond development. Duckweeds follow a peculiar growth strategy that makes them distinct from most higher plants: the fronds constantly produce clonal offspring that reaches maturity fast and starts producing their own offspring too. Duckweed colonies are, hence, clusters of fronds with different stages of maturity. This results in a dynamic system with both carbon sources (mature fronds with positive net assimilation) and carbon sinks (developing fronds with yet-low assimilating capacity) being present at the same time. Ambient temperature can influence overall carbon balance of such a colony in numerous ways, including the effect of thermal time on frond maturation, diverging temperature dependence of assimilation and respiration, and heat sensitivity of developmental and metabolic processes amongst others. We found that the young fronds of *La. punctata* and *L. gibba* had lower ChlF efficiencies at 35 °C when compared to lower temperatures but the mature fronds had similar efficiencies. This might be a result of a “carry-over” effect whereby the mature fronds transferred from the stock cultures (produced and developed under optimum conditions) have a higher temperature resilience as compared to the younger fronds produced under elevated temperature. A similar pattern was reported by Peršić et al. (2025) who observed stronger decline in photosynthetic performance of first-generation *L. minor* daughter fronds under sulphur deficiency as compared to their mother fronds that displayed a more stable performance under deteriorating conditions. It was also interesting that similar ChlF efficiencies in the younger fronds in *L. minor* as compared to the mature fronds at 30 and 35 °C were associated with different ontogenetic behaviours. The younger fronds, in this case, increased their ChlF efficiency at 30 °C, but the mature fronds, on the other hand, dropped their efficiency at 35 °C. Therefore, it might be possible that the functioning of the younger *L. minor* fronds was promoted under 30 °C, but further increase in temperature to 35 °C was already detrimental to them.

Our results also show that the apical (distal) part of fronds reaches photosynthetic maturity first, and then maturation progressively spreads towards the base. The observed lower and distinct ChlF efficiencies around the fronds’ basal compared to the distal parts were also previously reported (Oláh et al. 2024; Xuan et al. 2025). This change, however, occurred gradually by increasing the temperature between 20 and 30 °C and significant differences could only be observed between 20 and 35 °C treatments. The proportion of fronds with shorter lengths also dominated at 35 °C which could be related to stress acclimation response of duckweed to higher temperatures (Potters et al. 2007). Reduced shoot growth under elevated temperatures in aquatic higher plants has also been reported. For example, lower biomass production and decreased branch length were reported in *Myriophyllum aquaticum* at 30 °C (Wang et al. 2024). Similarly, a decrease in leaf number and shoot biomass was reported under high temperature (27 °C in water) for the free-floating plant *Hydrocharis morsus-ranae* (Koleszár et al. 2024) and submerged aquatic plants *Myriophyllum spicatum* and *Vallisneria natans* L. (Koleszár et al. 2022; Zhou et al. 2023).

The results of this study showed that duckweed growth and photosynthetic performance are slowed at higher temperatures. Different duckweed species, due to their inter-specific variations, trade-offs and intrinsic growth characteristics, respond differently upon exposure to changes in ambient temperatures (Mascheretti et al. 2026). Hence, climatic shifts could halt or favour the distribution of a specific duckweed species or clone and can alter the overall duckweed biodiversity. Our study also pointed out within-frond changes in photosynthetic efficiencies representing cellular-level adaptation in response to different temperature regimes.

### Global implications, limitations and outlook

Duckweeds are typically considered stress-tolerant and highly adaptive to variable growth conditions (Acosta et al. 2021). Despite being capable of maintaining relatively stable growth under sub-optimal conditions, changes in ambient temperatures, especially above 30 °C, can cause growth disruption and lower photosynthetic performance in certain species. These changes not only affect overall productivity of the plants but also their biochemical composition. Higher temperatures resulting in lower protein yields can make duckweeds lose their potential as an alternative protein crop (Islam et al. 2025). In addition, the effects of climate change on plants are widely modelled and incorporated into governance reports, such as IPCC (2023), based on terrestrial plant species. However, these effects on aquatic plants are often variable as shown by the results of the current study. Therefore, terrestrial model plants might not fully represent responses of aquatic plants. This study, hence, underpins the need for recognising aquatic plant models such as Lemnaceae to be included in climate change models.

It should be noted, however, that our experimental setup did not match any specific natural climate scenario, hence limiting generalisation of the presented results. Duckweeds are known to be less responsive to photoperiod, and display vigorous growth even under constant illumination when light intensity is non-saturating (Yin et al. 2015; Demmig-Adams et al. 2022). Yet, circadian rhythms can influence ontogenetic development of single fronds and, thus, affect species-specific patterns reported in this study. Similarly, light intensity, photoperiod and temperature have complex, interactive effects on plant developmental and metabolic processes, including such basic regulating mechanisms as the turnover of D1 protein of PSII, and carbon balance as a function of assimilation and respiration rates (Demmig-Adams et al. 2022; Pasos-Panqueva et al. 2024).

In addition, duckweed traits and responses are known to be clone-specific (Ziegler et al. 2015). It has been recently reported by Mascheretti et al. (2026) that growth responses of duckweed clones to different temperature regimes were divergent even within the same species. Caution should, therefore, be taken when generalising outcomes of single-clone studies.

Despite the above limitations, this study highlighted that temperature acclimation of duckweeds takes place at the level of fronds and ontogenetic gradients within fronds, and this can help in explaining colonisation and competitive abilities of species under a warming climate. Whilst characterisation of response patterns within fronds can be achieved using modulated fluorometry, this is currently not easily achieved for other relevant markers, including antioxidant enzymes, heat shock proteins, as well as expression of crucial regulatory genes. In this sense, the development of fractionated analyses on frond-cohorts with different maturity levels (Peršić et al. 2025), or single-cell sequencing of different frond regions (Abramson et al. 2022; Denyer et al. 2024; Yuan et al. 2025) offer scope to facilitate future deeper understanding of duckweed acclimation to elevated temperatures.

## Conclusion

This study proved that different species had variable growth, photosynthetic responses and frond-level adaptations under different temperatures. Higher temperatures generally resulted in slower growth, smaller fronds, decrease in overall photosynthetic performance and delayed frond maturity. This suggests that, despite the presence of high inter-specific variability across the duckweed family, a rise in surface temperatures in freshwaters could negatively impact most of the duckweed species and alter their competitiveness. These findings also highlight the importance of disentangling duckweed responses to elevated temperatures at frond level.

## Supplementary Information

Below is the link to the electronic supplementary material.

Supplementary file1 (DOCX 1709 KB)

## Data Availability

The datasets generated during and/or analysed during the current study are available from the corresponding author on reasonable request.
