# Supplementary material for: Effect of increasing temperatures on duckweed growth, photosynthetic performance and frond ontogenesis
Source: Plant Cell Rep. 2026 Aug 18;45(9):249. doi: 10.1007/s00299-026-03943-1 (PMC13486108; doi:10.1007/s00299-026-03943-1)
Supplement: Supplementary file 1 [file 299_2026_3943_MOESM1_ESM.docx]

**Supplementary material**

**Article title:** Effect of increasing temperatures on duckweed growth, photosynthetic performance and frond ontogenesis

**Journal:** Plant Cell Reports

**Authors:** Muhammad Irfan^1,2^, Muhammad Adil Chaudhry^2^, Manuela Bog^3^, Sándor Szabó^4^, Ilona Mészáros^2^, Marcel A.K. Jansen^1^, Viktor Oláh^2*^

**Authors affiliations:**

^1^School of Biological, Earth and Environmental Sciences, Sustainability Institute, University College Cork, Distillery Fields, North Mall, T23 TK30 Cork, Ireland

^2^Department of Botany, Institute of Biology and Ecology, Faculty of Science and Technology, University of Debrecen, Egyetem square 1, H-4032 Debrecen, Hungary

^3^Institute of Botany and Landscape Ecology, University of Greifswald, Soldmannstr. 15, 17489 Greifswald, Germany

^4^Department of Biology, Institute of Environmental and Natural Sciences, University of Nyíregyháza, H-4401 Nyiregyhaza, Hungary

^*^Correspondence: [olahviktor@unideb.hu](mailto:olahviktor@unideb.hu)

**Table S1:** Two-way ANOVA results of species, temperature, and interaction effects on growth- (RGR, Avg. Colony size and Frond area) and ChlF-based (F_v_/F_m_ and Y(II)) parameters.

| **Parameter** | **Variables** | **Df** | **Sum Sq** | **Mean Sq** | **F-value** | **p-value** | **η^2^** | **ω^2^** | **ε^2^** | **Cohen’s *f*** |
| --- | --- | --- | --- | --- | --- | --- | --- | --- | --- | --- |
| RGR | Species | 4 | 0.0486 | 0.0122 | 8.338 | 7.60E-06 | 0.028 | 0.024 | 0.024 | 0.578 |
|  | Temperatures | 3 | 1.216 | 0.4053 | 278.107 | < 2E-16 | 0.693 | 0.69 | 0.69 | 2.888 |
|  | Species*Temperatures | 12 | 0.3453 | 0.0288 | 19.742 | < 2E-16 | 0.197 | 0.187 | 0.187 | 1.539 |
|  | Residuals | 100 | 0.1458 | 0.0015 |  |  |  |  |  |  |
| Avg. Colony size | Species | 4 | 96.25 | 24.063 | 22.145 | 4.11E-13 | 0.288 | 0.274 | 0.275 | 0.941 |
|  | Temperatures | 3 | 79.37 | 26.457 | 24.349 | 6.54E-12 | 0.238 | 0.227 | 0.228 | 0.855 |
|  | Species*Temperatures | 12 | 49.66 | 4.138 | 3.809 | 8.90E-05 | 0.149 | 0.109 | 0.11 | 0.676 |
|  | Residuals | 100 | 108.66 | 1.087 |  |  |  |  |  |  |
| Avg. Frond area | Species | 4 | 2263.7 | 565.9 | 434.299 | < 2E-16 | 0.886 | 0.883 | 0.884 | 4.168 |
|  | Temperatures | 3 | 88.4 | 29.5 | 22.617 | 2.94E-11 | 0.035 | 0.033 | 0.033 | 0.824 |
|  | Species*Temperatures | 12 | 72.7 | 6.1 | 4.647 | 5.86E-06 | 0.028 | 0.022 | 0.022 | 0.747 |
|  | Residuals | 100 | 130.3 | 1.3 |  |  |  |  |  |  |
| F_v_/F_m_ | Species | 4 | 0.102 | 0.0255 | 11.54 | 9.64E-08 | 0.13 | 0.119 | 0.119 | 0.679 |
|  | Temperatures | 3 | 0.2092 | 0.06974 | 31.565 | 1.93E-14 | 0.268 | 0.258 | 0.259 | 0.973 |
|  | Species*Temperatures | 12 | 0.2494 | 0.02078 | 9.406 | 6.26E-12 | 0.319 | 0.284 | 0.285 | 1.062 |
|  | Residuals | 100 | 0.221 | 0.00221 |  |  |  |  |  |  |
| Y(II) | Species | 4 | 0.665 | 0.01662 | 20.583 | 2.14E-12 | 0.121 | 0.115 | 0.115 | 0.907 |
|  | Temperatures | 3 | 0.338 | 0.11267 | 139.511 | < 2E-16 | 0.615 | 0.609 | 0.61 | 2.046 |
|  | Species*Temperatures | 12 | 0.0647 | 0.00539 | 6.677 | 1.15E-08 | 0.118 | 0.1 | 0.1 | 0.895 |
|  | Residuals | 100 | 0.0808 | 0.00081 |  |  |  |  |  |  |

**Table S2:** Basic summary statistics for Kernel density plots **(Fig. 2)** showing minimum, maximum, mean and median length of fronds for tested duckweed species *S. polyrhiza*, *La. punctata*, *L. gibba*, *L. minor* and *L. minuta* under different ambient temperatures.

| Species | Temperature | Total No. of fronds | Min. length (mm) | Max. length (mm) | Mean length (mm) | Median length (mm) | Skewness |
| --- | --- | --- | --- | --- | --- | --- | --- |
| *S. polyrhiza* | 20 °C | 71 | 1.30 | 8.04 | 5.05 | 5.65 | -0.65 |
|  | 25 °C | 203 | 1.09 | 8.91 | 5.06 | 5.43 | -0.31 |
|  | 30 °C | 273 | 0.65 | 8.04 | 4.52 | 4.78 | -0.28 |
|  | 35 °C | 121 | 1.09 | 6.96 | 4.12 | 4.35 | -0.41 |
| *La. punctata* | 20 °C | 73 | 0.87 | 5.00 | 3.49 | 3.70 | -1.07 |
|  | 25 °C | 201 | 0.65 | 6.30 | 3.39 | 3.70 | -0.27 |
|  | 30 °C | 167 | 0.43 | 5.21 | 2.74 | 3.2 | -0.41 |
|  | 35 °C | 56 | 0.87 | 4.35 | 3.03 | 3.48 | -0.99 |
| *L. gibba* | 20 °C | 100 | 1.09 | 5.43 | 3.49 | 3.70 | -0.59 |
|  | 25 °C | 231 | 1.09 | 6.09 | 3.43 | 3.48 | -0.15 |
|  | 30 °C | 317 | 0.65 | 5.22 | 2.78 | 2.83 | 0.03 |
|  | 35 °C | 58 | 1.30 | 4.78 | 3.11 | 2.93 | 0.01 |
| *L. minor* | 20 °C | 107 | 1.30 | 6.09 | 4.05 | 4.38 | -0.43 |
|  | 25 °C | 207 | 1.09 | 6.30 | 3.66 | 3.48 | 0.004 |
|  | 30 °C | 123 | 0.87 | 6.09 | 3.47 | 3.48 | -0.15 |
|  | 35 °C | 65 | 1.09 | 5.87 | 3.69 | 3.91 | -0.40 |
| *L. minuta* | 20 °C | 148 | 0.65 | 2.83 | 1.87 | 1.96 | -0.59 |
|  | 25 °C | 189 | 0.43 | 3.04 | 1.76 | 1.96 | -0.17 |
|  | 30 °C | 221 | 0.43 | 2.61 | 1.65 | 1.74 | -0.50 |
|  | 35 °C | 36 | 0.87 | 2.17 | 1.58 | 1.74 | -0.51 |

**Table S3.** Parameters and correlation coefficients of the ordinary least squares linear regression models for F_v_/F_m_ and Y(II) as the function of frond length for tested duckweed species *S. polyrhiza*, *La. punctata*, *L. gibba*, *L. minor* and *L. minuta* under different ambient temperatures. Regression models with significant (p < 0.05) fit are highlighted by bold letters. Different lower-case letters (a, b, c) denote statistically significant differences (p < 0.05) of the regression model slopes for the same species across different temperatures, whereas different capital letters (A, B, C) denote statistically significant differences (p < 0.05) among different species at the same temperature, respectively. Note: slopes of non-significantly (p > 0.05) fitting regression models were excluded from comparisons, hence lack lower case letters and capitals.

| **Parameter** | **Species** | **Temperature** | **a (slope)** | **b (intercept)** | **Pearson's r** | **p (uncorrelated)** |
| --- | --- | --- | --- | --- | --- | --- |
|  | *S. polyrhiza* | 20 °C | **0.0168bB** | **0.6466** | **0.6001** | **<0.001** |
|  |  | 25 °C | **0.0323aAB** | **0.5418** | **0.7190** | **<0.001** |
|  |  | 30 °C | **0.0214bB** | **0.6018** | **0.5067** | **<0.001** |
|  |  | 35 °C | **0.0165bC** | **0.6864** | **0.6822** | **<0.001** |
|  | *La. punctata* | 20 °C | **0.0234bB** | **0.6601** | **0.6806** | **<0.001** |
|  |  | 25 °C | **0.0242bB** | **0.6660** | **0.5076** | **<0.001** |
|  |  | 30 °C | **0.0230bB** | **0.6874** | **0.4814** | **<0.001** |
|  |  | 35 °C | **0.0840aB** | **0.3848** | **0.7932** | **<0.001** |
| **F_v_/F_m_** | *L. gibba* | 20 °C | **0.0402bA** | **0.6034** | **0.7674** | **<0.001** |
|  |  | 25 °C | **0.0333bAB** | **0.6170** | **0.6246** | **<0.001** |
|  |  | 30 °C | **0.0296bB** | **0.6336** | **0.5275** | **<0.001** |
|  |  | 35 °C | **0.1427aA** | **0.1368** | **0.7337** | **<0.001** |
|  | *L. minor* | 20 °C | **0.0206bB** | **0.6368** | **0.7083** | **<0.001** |
|  |  | 25 °C | **0.0382aA** | **0.5583** | **0.7714** | **<0.001** |
|  |  | 30 °C | -0.0003 | 0.7676 | -0.0116 | 0.935 |
|  |  | 35 °C | 0.0005 | 0.6313 | 0.0082 | 0.963 |
|  | *L. minuta* | 20 °C | **0.0359aAB** | **0.6156** | **0.3824** | **<0.001** |
|  |  | 25 °C | **0.0411aA** | **0.6115** | **0.3720** | **<0.001** |
|  |  | 30 °C | **0.0519aA** | **0.6279** | **0.5810** | **<0.001** |
|  |  | 35 °C | -0.0035 | 0.4990 | -0.0060 | 0.976 |
|  | *S. polyrhiza* | 20 °C | **0.0269abAB** | **0.4821** | **0.6971** | **<0.001** |
|  |  | 25 °C | **0.0321aA** | **0.4470** | **0.6828** | **<0.001** |
|  |  | 30 °C | **0.0324aB** | **0.4420** | **0.6057** | **<0.001** |
|  |  | 35 °C | **0.0132bB** | **0.4781** | **0.3085** | **<0.05** |
|  | *La. punctata* | 20 °C | **0.0357bA** | **0.5300** | **0.8094** | **<0.001** |
|  |  | 25 °C | **0.0333bA** | **0.5437** | **0.7709** | **<0.001** |
|  |  | 30 °C | **0.0269bB** | **0.5880** | **0.7672** | **<0.001** |
|  |  | 35 °C | **0.0915aA** | **0.2317** | **0.7298** | **<0.001** |
| **Y(II)** | *L. gibba* | 20 °C | **0.0185bB** | **0.5851** | **0.4276** | **0.0035** |
|  |  | 25 °C | **0.0433bA** | **0.4753** | **0.5431** | **<0.001** |
|  |  | 30 °C | **0.0345bB** | **0.5306** | **0.6009** | **<0.001** |
|  |  | 35 °C | **0.1290aA** | **0.0155** | **0.7684** | **<0.001** |
|  | *L. minor* | 20 °C | **0.0168bB** | **0.5542** | **0.5356** | **<0.001** |
|  |  | 25 °C | **0.0429aA** | **0.4589** | **0.7445** | **<0.001** |
|  |  | 30 °C | 0.0031 | 0.6600 | 0.1253 | 0.344 |
|  |  | 35 °C | 0.0032 | 0.5737 | 0.0491 | 0.785 |
|  | *L. minuta* | 20 °C | **0.0293bAB** | **0.5382** | **0.4269** | **<0.001** |
|  |  | 25 °C | **0.0433bA** | **0.5369** | **0.6156** | **<0.001** |
|  |  | 30 °C | **0.0586aA** | **0.5262** | **0.7875** | **<0.001** |
|  |  | 35 °C | 0.0777 | 0.3669 | 0.2744 | 0.334 |

**Figure S1:** Within-frond patterns in dark-adapted photosynthetic efficiency F_v_/F_m_ of the tested duckweed species **a-b)** *S. polyrhiza*, **c-d)** *La. punctata*, **e-f)** *L. gibba*, **g-h)** *L. minor* and **i-j)** *L. minuta* for different frond ontogenetic stages under 25 and 30 °C ambient temperatures. The interpolation plots represent pixel-by-pixel F_v_/F_m_ values of different frond sizes along the horizontal axis and within-frond measured position from the frond base along the vertical axis. Black dots denote positions of the actually measured pixels along the transects. Note: The color gradients were set separately for each plot.

**Figure S2:** Within-frond patterns in light-adapted actual photosynthetic efficiency Y(II) of the tested duckweed species **a-b)** *S. polyrhiza*, **c-d)** *La. punctata*, **e-f)** *L. gibba*, **g-h)** *L. minor* and **i-j)** *L. minuta* for different frond ontogenetic stages under 25 and 30 °C ambient temperatures. The interpolation plots represent pixel-by-pixel F_v_/F_m_ values of different frond sizes along the horizontal axis and within-frond measured position from the frond base along the vertical axis. Black dots denote positions of the actually measured pixels along the transects. Note: The color gradients were set separately for each plot.
